# Cardiovascular risk factors and 30-year cardiovascular risk in homeless adults with mental illness

**DOI:** 10.1186/s12889-015-1472-4

**Published:** 2015-02-23

**Authors:** Agnes Gozdzik, Roxana Salehi, Patricia O’Campo, Vicky Stergiopoulos, Stephen W Hwang

**Affiliations:** Centre for Research on Inner City Health, Li Ka Shing Knowledge Institute of St. Michael’s Hospital, 30 Bond Street, Toronto, ON M5B 1 W8 Canada; Dalla Lana School of Public Health, University of Toronto Health Sciences Building, 6th floor, 155 College Street, Toronto, ON M5T 3 M7 Canada; Department of Psychiatry, University of Toronto, 250 College Street, 8th Floor, Toronto, ON M5T 1R8 Canada; Division of General Internal Medicine, Department of Medicine, University of Toronto, Toronto, ON Canada

**Keywords:** Cardiovascular diseases, Cardiovascular risk factors, Homeless persons, Mental illness

## Abstract

**Background:**

Cardiovascular disease (CVD) is a leading cause of death among homeless people. This study examines CVD risk factors and 30-year CVD risk in a population of homeless individuals with mental illness.

**Methods:**

CVD risks factors were assessed in 352 homeless individuals with mental illness in Toronto, Canada, at the time of their enrollment in the At Home/Chez Soi Project, a randomized trial of a Housing First intervention. The 30-year risk for CVD (coronary death, myocardial infarction, and fatal or nonfatal stroke) was calculated using published formulas and examined for association with need for mental health services, diagnosis of psychotic disorder, sex, ethnicity, access to a family physician and diagnosis of substance dependence.

**Results:**

The 30-year CVD risk for study participants was 24.5 ± 18.4%, more than double the reference normal of 10.1 ± 7.21% (difference = −13.0% 95% CI −16.5% to −9.48%). Univariate analyses revealed 30-year CVD risk was greater among males (OR 3.99, 95% CI 2.47 to 6.56) and those who were diagnosed with substance dependence at baseline (OR 1.94 95% CI 1.23 to 3.06) and reduced among those who were non-white (OR 0.62 95% CI 0.39 to 0.97). In adjusted analyses, only male sex (OR 4.71 95% CI 2.76 to 8.05) and diagnosis of substance dependence (OR 1.78 95% CI 1.05 to 3.00) remained associated with increased CVD risk.

**Conclusions:**

Homeless people with mental illness have highly elevated 30-year CVD risk, particularly among males and those diagnosed with substance dependence. This study adds to the literature by reporting on CVD risk in a particularly vulnerable population of homeless individuals experiencing mental illness, and by using a 30-year CVD risk calculator which provides a longer time-frame during which the effect of modifiable CVD risk factors could be mitigated.

**Trial registration:**

Current Controlled Trials ISRCTN42520374

**Electronic supplementary material:**

The online version of this article (doi:10.1186/s12889-015-1472-4) contains supplementary material, which is available to authorized users.

## Background

Homeless individuals experience high rates of morbidity and mortality [1-5], as well as many barriers to accessing appropriate health care [6-8]. Cardiovascular disease (CVD) is a leading cause of death among people experiencing homelessness [9-12]. Among homeless men and women in Boston aged 45–64 years old, mortality from CVD was 3.5 and 3.0 times higher, respectively, than in the general population [12].

The increased CVD risk among homeless individuals likely results from the interaction of traditional cardiovascular risk factors and other risk factors associated with homelessness. Several studies report that homeless people have an increased prevalence of traditional CVD risk factors, including smoking [13,14] and undiagnosed or poorly controlled hypertension, diabetes, and hypercholesteremia [13-16]. Substance use [17-20] and mental illness [21,22] are both associated with increased risk of CVD and found at disproportionately high rates among homeless people compared to the general population [20]. Use of anti-psychotic medication, particularly “atypical” or second-generation antipsychotics, has also been associated with cardiovascular risk factors, such as diabetes, dyslipidaemia and obesity [23-25]. Finally, low socioeconomic status (SES) and chronic stress are ubiquitous among the homeless population, and both have known associations with increased CVD risk [26,27].

In order to further expand the literature on CVD risk among homeless individuals with mental illness, this study first examines the prevalence of CVD risk factors of participants enrolled in the Toronto site of the At Home/Chez Soi project, a randomized trial evaluating a Housing First intervention among homeless adults with mental illness. Secondly, we expand upon these observations by assessing the 30-year CVD risk in this population: while previous studies have examined the 10-year coronary heart disease (CHD) risk among homeless populations [14,28], 30-year CVD estimates allows for a longer time-frame during which the effect of modifiable CVD risk factors could be mitigated. Finally, both prevalence of CVD risk factors and 30-year CVD risk are assessed for associations with need for mental health services, diagnosis of a psychotic disorder, sex, ethnicity, access to a family physician and diagnosis of a substance use disorder.

## Methods

### Study population

This study uses data collected from participants recruited at the Toronto site of the At Home/Chez Soi project, a randomized controlled trial of the Housing First model for homeless individuals with mental illness, conducted in five cities in Canada (Vancouver, Winnipeg, Toronto, Montreal and Moncton). The Housing First model is a consumer-driven intervention which provides immediate or rapid provision of permanent housing as the first step to recovery, in conjunction with ongoing mental health supports and case management [29-32]. Unlike traditional interventions, Housing First does not require participants to accept psychiatric treatment or abstain from substance use as a condition for housing. Detailed descriptions of the project, including the Toronto site, have been published previously [33,34].

Briefly, Toronto At Home/Chez Soi participants were recruited via referrals from a network of mental health and homelessness agencies in the city, including hospitals, mental health teams and shelters, and were assessed for eligibility by an intake coordinator. Eligibility criteria for the study were: 1) age 18 years or over; 2) absolute homelessness or precarious housing (see Additional file 1: Tables S1); and 3) mental illness, with or without coexisting substance use disorder, based on DSM-IV criteria using the MINI International Neuropsychiatric Interview (MINI) [29,30]. Exclusion criteria included: 1) being a current client of an assertive community treatment (ACT) or intensive case management program (ICM); and 2) lack of legal residence status in Canada. Participants could not be current ACT or ICM clients because these services were provided to the intervention group and their effectiveness was under evaluation in the trial. Legal status in Canada was necessary to qualify for government income assistance, which was sought for eligible participants: in the Housing First model, up to 30% of participant income could be used to offset the cost of housing [31]. Participant baseline measures took place from October 2009 to June 2011.

Because this study focuses only on baseline interview measures, participant randomization and receipt of services was not relevant to our analysis, and participants from both treatment and usual care groups are included in all analyses.

Individuals were excluded from this analysis if they had characteristics that precluded calculation of 30-year CVD risk: 1) established cardiovascular disease at the time of enrolment; 2) diagnosis of cancer; or 3) age ≤20 years or >60 years old [32]. Individuals were also excluded if they did not have complete data for the variables used in the 30-year CVD risk calculator (see Additional file 1: Table S3).

All participants provided written informed consent. The study was approved by the Research Ethics Board of St. Michael’s Hospital and was registered with the International Standard Randomized Control Trial Number Register (ISRCTN42520374).

### Measures

Study participants completed baseline questionnaires and physical measurements. Blood samples were not collected due to logistic challenges and concerns regarding the willingness of individuals to participate in the study if such a request were made. As a result, lipid profiles of participants were not obtained.

### Self-report data

Self-reported data were obtained on demographic characteristics, presence of chronic diseases and access to health care [33,34].

### Substance Use

We report specifically on smoking, alcohol, any cocaine (including crack cocaine) and marijuana use in the past month, because of the high prevalence of use of these substances (>10%) in our sample (data not shown). Furthermore, both smoking [14] and cocaine [19] have known associations with CVD risk.

### Perceived stress

We used the 10-item Perceived Stress Scale to assess participant perceived stress during the past month. This instrument uses a 5-point Likert scale from 0 (“never”) to 4 (“often”) to rate frequency of feelings regarding life situations [35]. The values on four positive questions were reversed (items 3, 5, 7, 8) and a total score was tabulated (ranging from 0 to 40), with higher total scores indicating higher perceived stress.

### MINI International Neuropsychiatric Interview 6.0 (MINI 6.0)

The MINI 6.0 structured diagnostic interview was used to determine the presence of mental disorders at the time of study entry [29]. Individuals were eligible for the study if at study entry, they demonstrated the following current diagnoses: 1) major depressive episode; 2) manic or hypomanic episode; 3) post-traumatic stress disorder; 4) panic disorder; 5) mood disorder with psychotic features; or 6) psychotic disorder [33]. The MINI has been validated against the Structure Clinical Interview for DSM Diagnoses (SCID-P) and the Composite International Diagnostic Interview for ICD-10 (CIDI) [29,30,36].

### Physical measures

Weight was measured using a portable digital scale (Conair Consumer Products, Inc.). Height was measured using a wall-mounted tape measure (Stanley Corp.). Waist and hip circumference were measured with a tape measure (Aemedic) and rounded to the nearest centimeter. Body mass index (BMI) was calculated as weight in kilograms divided by the height in meters squared (kg/m2) [34].

### Blood pressure

Blood pressure was measured 3 times in one arm using an automatic blood pressure monitor (LifeSource UA-767 plus) with the subject seated comfortably. Measurements were taken from the right arm whenever possible, with readings taken at least 30 seconds apart. Mean systolic and diastolic pressures were calculated for each participant. Blood pressure was classified as normal (<120 mmHg systolic and <80 mmHg diastolic), pre-hypertension (120 to 139 mmHg systolic or 80 to 89 mmHg diastolic), or hypertension (≥140 mmHg systolic or ≥90 mmHg diastolic), according to the recommendations of the Joint National Committee on Prevention, Detection, Evaluation and Treatment of Blood Pressure (JNC) 7 [37]. Hypertension can be further classified as stage 1 or stage 2, but these two categories were merged due to the small number of participants meeting criteria for hypertension. Because some participants reported a previous diagnosis of hypertension and these individuals’ blood pressure measurements may have reflected treated values, we conducted separate analyses in which these individuals were excluded. This exclusion did not result in any substantial changes in our findings, and thus the results of these analyses are not shown.

### 30-year CVD risk

We calculated the 30-year risk of CVD using a formula derived from the Framingham study [32]. The CVD risk calculation is based on age, sex, mean systolic pressure, presence of diabetes, hypertension treatment, smoking status, and BMI [32]. The CVD risk calculator generates 30-year risk scores for two outcomes, “hard CVD” and “full CVD”. Hard CVD consists of coronary death, myocardial infarction, and fatal or nonfatal stroke. Full CVD includes all hard CVD outcomes plus coronary insufficiency, angina pectoris, transient ischemic attack, intermittent claudication, and congestive heart failure [38]. We have elected to focus on hard CVD due to its clarity and clinical relevance. All analyses presented here pertain to the outcome of hard CVD, which is simply referred to as CVD.

The 30-year CVD risk is classified as low risk (<12%), intermediate risk (≥12% and <40%), or high risk (≥40%) [32]. The CVD risk calculator also provides a “normal” CVD risk score for each individual based on the individual’s age and sex and the following idealized risk factor profile: 1) non-smoker; 2) non-diabetic; 3) not treated for blood pressure; 4) systolic blood pressure of 125 mmHg; and 5) BMI of 22.5 (M.J. Pencina, 2012, personal communication).

### Predictors of 30-year CVD risk

We examined the predictors listed below for their association with the prevalence of CVD risk factors and 30-year CVD risk levels (low vs. intermediate/high):

#### 1) Need level for mental health services

At the time of study enrolment, participants were stratified into one of two need level groups (high or low) for mental health services using an algorithm based on functioning and service use, including baseline MINI diagnoses, scores on the Multnomah Community Ability Scale (MCAS) [39], as well as prior history of psychiatric hospitalization and arrests (see Additional file 1: Table S2) [33].

#### 2) Diagnosis of psychosis

Prior to study enrolment, participants were screened for presence of a diagnosis of a Current Psychotic Disorder, using the MINI (see above). This diagnosis was added to the model as a CVD predictor due to the known association between increased CVD risk factors and use of anti-psychotic medication [24].

#### 3) Sex

Self-reported gender was collected at the baseline interview. 30-year CVD risk scores could only be calculated for participants indicating male or female sex, as a result, participants who indicated other gender(s) were not included in this study.

#### 4) Ethnicity

Participant ethnicity was based on Statistics Canada definitions [40,41]. Participants who self-identified as belonging to the following ethnic or cultural identities were considered “Ethno-Racial” (previously identified as “Visible Minority”): East Asian (e.g. China, Japan, Korea), South Asian (e.g. India, Pakistan, Sri Lanka), South East Asian (e.g. Malaysia, Philippines, Vietnam), Black African (e.g. Ghana, Kenya, Somalia), Black Canadian/American, Black Caribbean Region (e.g. Jamaica, Trinidad, Tobago), Latin American (e.g. Argentina, Chile, Costa Rica), Indian-Caribbean (e.g. Guyana with origins in India), Middle Eastern (e.g. Egypt, Iran, Israel, Palestine) or mixed background (that included at least one of the ethnic groups listed above). Participants who indicated White European or White Canadian ethnicity were considered “Non Ethno-Racial”. Participants who indicated membership in communities of Aboriginal peoples were considered “Aboriginal” but this group was excluded from analyses by ethnicity due to its small size (N = 16).

#### 5) Access to family physician

Participants were asked to report if they had access to a family physician at the baseline interview.

#### 6) Diagnosis of substance dependence

Prior to study enrolment, participants were screened for presence of Current Substance Dependence using the MINI (see above).

### Statistical analysis

Descriptive statistics were calculated for each baseline measure and 30-year CVD risk scores. T-tests, Mann–Whitney U tests, Wilcoxon Signed-Rank Test and chi-square tests, as appropriate, were used to test for the association of need level, diagnosis of psychotic disorder, sex, ethnicity, access to family physician and diagnosis of substance dependence with CVD risk factors. Finally, univariate and multivariate analyses were conducted using binomial logistic regression, to identify the individual contribution of each of these variables (need level, diagnosis of psychotic disorder, sex, ethnicity, access to family physician and diagnosis of substance dependence) to 30-year CVD risk level category (low or increased risk). We collapsed the two higher risk categories (intermediate and high) due to the considerably smaller proportion of individuals who had the high risk category. All regression models were assessed for multicollinearity. Analyses were performed using IBM SPSS Statistics version 21 with the significance level set at 0.05.

## Results

### Study participants

A total of 575 participants completed the baseline interview at the Toronto site of the At Home/Chez Soi project. Of this number, we excluded 59 (10.3%) individuals who reported a pre-existing history of cardiovascular disease (18 with heart disease, 15 with stroke, 15 with other CVD, and 11 with more than one CVD); 8 individuals who reported having cancer; and 22 individuals who were aged <20 years (N = 7) or ≥60 years (N = 15). An additional 134 individuals were excluded because they lacked complete data required to calculate 30-year CVD risk. The final sample size for this study was therefore 352. Compared to excluded participants (N = 223), included participants (N = 352) were less likely to be born in Canada (51.0% vs. 59.6%, P = 0.043). Excluded compared to included participants were also older (41.6 vs. 38.7 years, P = 0.016), had a longer period of total lifetime (6.0 vs. 4.7 years, P = 0.003) and longest single episode of homelessness (3.2 vs. 2.7 years, P = 0.022). A greater number of excluded vs. included participants reported no access to medical care when it was required (44.6% vs. 34.9%, P = 0.021).

The final study sample (N = 352) consisted largely of ethno-racial (64.6%), male (71.0%) participants, of whom more than a third had a diagnosis of psychotic (38.1%) and/or depressive (36.9%) disorder, with nearly two-thirds reporting having access to a family physician (65.0%). Mean age among the sample was 38.7 ± 10.6 years however, females participant were younger than male counterparts (36.7 ± 11.1 vs. 39.5 ± 10.4 years, P = 0.026; difference = −2.76 95% CI −5.21 to 0.32). The characteristics of these participants are shown in Table 1.

**Table 1:** **Participant characteristics at enrollment at the Toronto site of the At Home/Chez Soi study**

**Characteristic** ^**a**^	**Total sample (N=352)** ^**b**^
**Age, mean (SD), y**	38.7 ± 10.6
**Gender**	
Female	102 (29.0)
Male	250 (71.0)
**Country of birth**	
Canada	179 (51.0)
Other	172 (49.0)
**Ethnic Background** ^c^	
Ethno-Racial	217 (64.6)
Non Ethno-Racial	119 (35.4)
**Current Housing Status**	
Absolutely homeless	331 (94.0)
Precariously housed	21 (6.0)
**Total length of homelessness lifetime, mean (SD), y**	4.76 ± 5.82
**Longest period of homeless, mean (SD), y**	2.74 ± 4.43
**Written documentation of mental illness**	105 (30.3)
**MINI Results** ^d^	
Depressive Episode	130 (36.9)
Manic/Hypomanic Episode	39 (11.1)
Post-Traumatic Stress Disorder	79 (22.4)
Panic Disorder	50 (14.2)
Mood Disorder with Psychotic Features	65 (18.5)
Psychotic Disorder	134 (38.1)
Suicidality	231 (65.6)
Alcohol Dependence	101 (28.7)
Alcohol Abuse	44 (12.5)
Substance Dependence	128 (36.4)
Substance Abuse	32 (9.1)
**Access to family physician**	227 (65.0)
No access to medical care when it was required^f^	123 (34.9)

^a^N(%), unless otherwise noted.

^b^Data missing for the following characteristics:, Total Length of Homelessness (N = 6) Longest Period of Homelessness (N = 3), Years of School Completed (N = 2), Written Documentation of Mental Illness (N = 5), Has family physician (N = 2).

^c^Individuals who self-identified as not belonging to either an ethno-racial or non ethno-racial group (e.g. Aboriginals) were excluded from this analysis. As a result the total N for the Ethnic Background variable is 336.

^d^The MINI was administered at study entry, and diagnoses correspond to a “current” diagnosis at that point.

^e^Suicidality includes individuals who indicated low, moderate and high levels of suicidality.

^f^“No access” means the participant had need of healthcare but did not have access to healthcare.

### Prevalence of CVD risk factors

The prevalence of hypertension and diabetes among participants was 16.2% and 7.7%, respectively. More than two-thirds of participants were daily or occasional smokers (69.6%). In the past 30 days, nearly half (47.7%) had used alcohol, while more than a third (35.6%) used marijuana and one-fifth (19.9%) indicated cocaine use. Participants reported a mean perceived stress score of 22.0 ± 8.44. Mean participant BMI was 26.46 ± 5.93, mean waist circumference was 92.6 ± 14.5 cm, while the mean waist-to-hip ratio was 0.92 ± 0.07. Mean measured systolic and diastolic blood pressure were 121.3 ± 17.5 mmHg and 80.0 ± 11.8 mmHg, respectively, with nearly a third (33.3%) of participants having measured blood pressure values indicative of prehypertension, and nearly a quarter (23.6%) corresponding to hypertension [37].

Table 2 shows the distribution of CVD risk factors in the study population, stratified by need level for mental health services, diagnosis of psychotic disorder, sex, ethnicity, access to family physician and diagnosis of substance dependence. Compared to participants with moderate needs for mental health services, high needs participants had larger waist-to-hip ratios (0.94 vs. 0.92, P = 0.024; difference = 0.02 95% CI −0.008 to 0.03), greater alcohol use (56.0% vs. 44.0%, P = 0.038; difference = 11.9% 95% CI 0.007 to 23.1%), and lower perceived stress (19.8 vs. 22.8, P = 0.004; difference −3.02 95% CI −5.09 to −0.96)

**Table 2:** **Baseline cardiovascular risk factors and access to care measures, stratified by level of need for mental health services, diagnosis of psychotic disorder, sex, ethnicity, access to a family physician and diagnosis of substance dependence in a sample of participants from the Toronto site of the At Home/Chez Soi study**

	**Need level**		**Diagnosis of psychosis** ^**c**^		**Sex**		**Ethnicity** ^**d**^		**Has access to family physician** ^**e**^		**Diagnosis of substance dependence** ^**c**^	
	**Moderate**	**High**	**Yes**	**No**	**Male**	**Female**	**Ethno-racial**	**Non ethno-racial**	**Yes**	**No**	**Yes**	**No**
	**(N=243)**	**(N=109)**	**(N=134)**	**(N=218)**	**(N=250)**	**(N=102)**	**(N=217)**	**(N=119)**	**(N=227)**	**(N=122)**	**(N=128)**	**(N=224)**
**Self-reported variables** ^**b**^												
Hypertension	45 (18.5)	12 (11.0)	15 (11.2)	42 (19.3)*	41 (16.4)	16 (15.7)	33 (15.2)	18 (15.1)	45 (19.8)	12 (9.8)*	20 (15.6)	37 (16.5)
Diabetes	17 (7.0)	10 (9.2)	8 (6.0)	19 (8.7)	23 (9.2)	≤5 (≤5.0)	19 (8.8)	6 (5.0)	18 (7.9)	9 (7.4)	8 (6.3)	19 (8.5)
*Smoking*												
Daily/Occasionally	162 (66.7)	83 (76.1)	96 (71.6)	149 (68.3)	192 (76.8)	53 (52.0)***	138 (63.6)	95 (79.8)**	155 (68.3)	88 (72.1)	118 (92.2)	127 (56.7)***
Never	81 (33.3)	26 (23.9)	38 (28.4)	69 (31.7)	58 (23.2)	49 (48.0)	79 (36.4)	24 (20.2)	72 (31.7)	34 (27.9)	10 (7.8)	97 (43.3)
Any Alcohol Use	107 (44.0)	61 (56.0)*	54 (40.3)	114 (52.3)*	124 (49.6)	44 (43.1)	83 (38.2)	71 (59.7)***	107 (47.1)	58 (47.5)	88 (68.8)	80 (35.7)***
Any Cocaine Use^f^	44 (18.1)	26 (23.9)	21 (15.7)	49 (22.5)	49 (19.6)	21 (20.6)	28 (12.9)	36 (30.3)***	47 (20.7)	22 (18.0)	54 (42.2)	16 (7.1) ***
Marijuana Use	83 (34.3)	42 (38.5)	43 (32.1)	82 (37.8)	98 (39.2)	27 (26.7)*	70 (32.4)	46 (38.7)	79 (35.0)	44 (36.1)	84 (65.6)	41 (18.4)***
Perceived stress scale, Total scores, mean (SD)	22.8 (7.97)	19.8 (9.24)**	18.9 (7.95)	23.7 (8.22)***	21.9 (8.49)	22.1 (8.36)	21.5 (8.22)	22.9 (9.06)	22.5 (8.34)	20.7 (8.47)	24.0 (7.05)	20.8 (8.96)**
**Measured variables** ^**g**^												
BMI (kg/m2)	26.4 ± 6.20	26.5 ± 5.32	26.5 ± 5.65	26.5 ± 6.11	25.6 ± 4.73	28.5 ± 7.81**	26.7 ± 6.06	25.5 ± 5.06	26.8 ± 6.20	25.8 ± 5.38	26.0 ± 5.40	26.7 ± 6.21
Waist circumference, mean (SD), cm	91.9 ± 14.4	94.4 ± 14.6	92.9 ± 14.4	92.5 ± 14.6	92.9 ± 13.4	92.0 ± 17.0	92.3 ± 14.1	92.0 ± 14.8	93.2 ± 15.1	91.8 ± 13.3	92.5 ± 14.4	92.7 ± 14.6
Waist to hip ratio, mean (SD), cm	0.92 ± 0.07	0.94 ± 0.06*	0.93 ± 0.06	0.92 ± 0.07	0.94 ± 0.06	0.89 ± 0.06***	0.92 ± 0.07	0.92 ± 0.07	0.93 ± 0.07	0.92 ± 0.07	0.93 ± 0.07	0.92 ± 0.07
Blood Pressure^h^												
Systolic, mean (SD), mmHg	121.7 ± 17.8	120.4 ± 16.8	120.1 ± 17.9	122.1 ± 17.2	121.8 ± 16.6	120.1 ± 19.6	120.8 ± 17.6	121.6 ± 18.0	122.1 ± 18.8	119.8 ± 15.0	120.9 ± 18.4	121.5 ± 17.0
Diastolic, mean (SD), mmHg	80.0 ± 12.2	79.9 ± 11.0	79.6 ± 12.7	80.2 ± 11.3	79.1 ± 11.2	82.0 ± 13.0	80.0 ± 12.4	79.4 ± 10.7	80.3 ± 12.2	79.31 ± 11.1	78.5 ± 10.5	80.8 ± 12.4
*JNC7 Hypertension Categories* ^i^												
Normal/Prehypertension	185 (76.1)	84 (77.1)	102 (76.1)	167 (76.6)	199 (79.6)	70 (68.6)*	166 (76.5)	94 (79.0)	174 (76.7)	93 (76.2)	105 (82.0)	164 (73.2)
Hypertension I/II	58 (23.9)	25 (22.9)	32 (23.9)	51 (23.4)	51 (20.4)	32 (31.4)	51 (23.5)	25 (21.0)	53 (23.3)	29 (23.8)	23 (18.0)	60 (26.8)

*< 0.05; **< 0.01; ***< 0.001; P values represent group differences using T-tests, Fisher’s and chi-square test, as appropriate.

^a^Unless indicated, values correspond to N (%).

^b^Variables with missing values: Any Cocaine Use (N = 1).

^c^Presence of Psychotic Disorder and Substance Dependence were assessed by the MINI International Neuropsychiatric Interview at study entry.

^d^1 participant was excluded from this participant because they did not self-identify as either ethno-racial or non-ethnoracial.

^e^“No access” means the participant had need of healthcare but did not have access to healthcare.

^f^Any cocaine use includes both crack and cocaine use in the last 30 days.

^g^Measured variables with missing values: Waist Circumference (N = 9) and Waist-to-Hip (N = 9). All other variables had no missing values.

^h^Includes individuals who self-reported having hypertension.

^i^Based on measured hypertension, employing the Seventh Report of the Joint National Committee on Prevention, Detection, Evaluation and Treatment of High Blood Pressure [37].

.

Compared to individuals with a diagnosis of psychosis, those without this diagnosis had increased rate of hypertension (19.3% vs. 11.2%, P = 0.046; difference = 8.07% 95% CI 0.59 to 15.5%), higher alcohol use (52.3% vs. 40.3%, P = 0.029; difference = 12.0% 95% CI 1.40 to 22.6% and higher perceived stress scores (23.7 vs. 18.9, P < 0.001; difference = 4.83 95% CI 2.96 to 6.71).

Cardiovascular risk profiles differed significantly by sex. Higher prevalence rates were observed for males than females for smoking (76.8% vs. 52.0%, P < 0.001; difference 24.8% 95% CI 13.8 to 35.9%) and marijuana use (39.2% vs. 26.7%, P = 0.027; difference 12.5% 95% CI 1.93 to 23.0%). Females had significantly higher BMI values (28.5 vs. 25.6, P = 0.001; difference = 2.92 95% CI 1.28 to 4.57) and lower waist-to-hip ratios (0.89 vs. 0.94, P < 0.001; difference = −0.048 95% CI −0.063 to −0.033) than males. Blood pressure values in the hypertensive range were more likely among females compared to males (31.4% vs. 20.4%, P = 0.028; difference = 11.0% 95% CI 0.68 to 21.3%).

Ethnicity was associated with several cardiovascular risk factors. Smoking rates were higher among white individuals compared to individuals belonging to an ethno-racial group (79.8% vs. 63.6%, P = 0.002; difference = 16.2% 95% CI 6.66 to 25.9%). Alcohol use was also higher among white participants compared to those belonging to an ethno-racial group (59.7% vs. 38.2%, P < 0.001; difference = 21.4% 95% CI 10.5 to 32.3%), while use of cocaine was more than twice as high among white individuals compared to individuals from an ethno-racial group (30.3% vs. 12.9%, P < 0.001; difference = 17.3% 95% CI 7.97 to 26.7%).

Participants who reported access to a family physician indicated higher rates of self-reported hypertension (19.8%) compared to participants without a family physician (9.8%, P = 0.016; difference = 10.0% 95% CI 2.58 to 17.4%).

Rates of smoking, alcohol, cocaine and marijuana use were all significantly higher among those with a diagnosis of substance dependence than among those without this diagnosis (all P < 0.001; differences = 35.5% 95% CI 27.5 to 43.5%; 33.0% 95% CI 22.8 to 43.2%; 35.0% 95% CI 25.8 to 44.2%; and 47.2% 95% CI 37.6 to 56.9%, respectively; Table 2). Additionally, perceived stress scores were significantly higher among those with a diagnosis of substance dependence than among those without this diagnosis (24.0 vs. 20.8, P = 0.002; difference = 3.27 95% CI 1.46 to 5.07).

### 30-year CVD risk estimates

**Figure 1:**
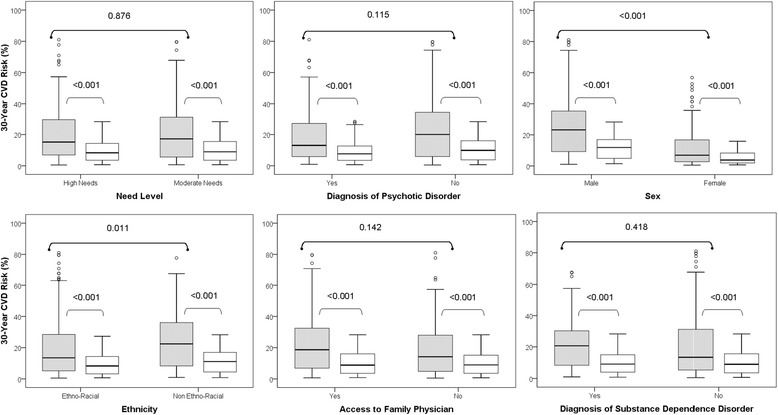
**Comparison between calculated and “normal” 30-year CVD risk scores (%), stratified by level of need for mental health services, diagnosis of psychotic disorder, sex, ethnicity, access to a family physician and diagnosis of substance dependence.** Significant differences were observed between calculated (shaded boxplots) and “normal” (clear boxplots) values for all comparisons (p < 0.001). For each boxplot, the top and bottom whiskers refer to the maximum and minimum values, respectively, while the top, middle and bottom lines of the box referring to the third quartile, median and first quartile, respectively. P-values from comparisons of the calculated CVD risk scores for the two categories within each predictor (shaded boxplots) are shown on the top bar.

The 30-year CVD risk for all participants was 24.5 ± 18.4%, more than twice as high as the reference normal risk of 10.1 ± 7.21% (P < 0.001; difference = 11.4% 95% CI 9.96 to 12.9%). Boxplots of the calculated 30-year CVD risk scores and corresponding reference “normal” scores, stratified by each of the six factors are shown in Figure 1.

The mean 30-year CVD risk score was significantly lower among females compared to males (12.2% vs. 25.2%, P < 0.001; difference = −13.0% 95% CI −16.5 to −9.48%) and among participants of ethno-racial ethnicity compared to those who were non ethno-racial (20.0% vs. 23.7%, P = 0.011; difference = −3.66 95% CI −7.72 to −0.41%). No significant differences were observed in 30-year CVD risk scores based on need level (P = 0.115), diagnosis of psychotic disorder (P = 0.876), access to family physician (P = 0.142) or diagnosis of substance dependence (P = 0.418) (Additional file 1: Table S4). There was a lack of association between the total perceived stress score and the 30-year CVD risk score (r = 0.084, P = 0.137).

When compared to idealized “normal” CVD risk scores, calculated CVD risk scores were significantly higher in all comparisons using a Wilcoxon Signed Rank Test (P < 0.001) (Figure 1).

**Figure 2:**
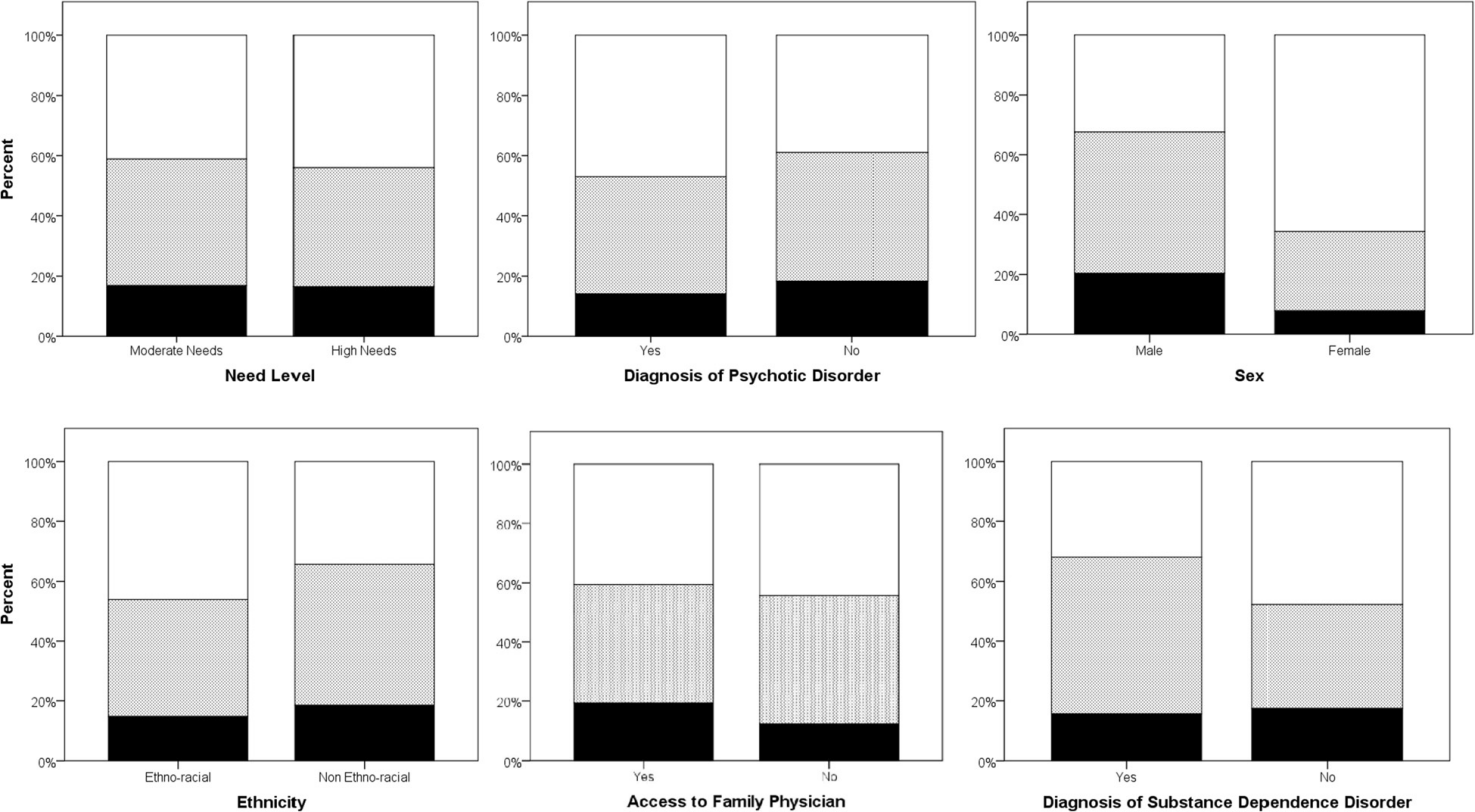
**30-year CVD risk categories, stratified by level of need for mental health services, diagnosis of psychotic disorder, sex, ethnicity, access to a family physician and diagnosis of substance dependence.** The proportion of individuals is given as the percentage of the total for each X axis category. The white, grey and black color of the bar corresponds to low, moderate and high CVD risk category, respectively.

Figure 2 shows the distribution of low (<12%), intermediate (≥12% and <40%) and high risk (≥40%) categories for 30-year CVD scores, stratified by need level, diagnosis of psychotic disorder, sex, ethnicity, access to family physician and diagnosis of substance dependence. CVD risk categories were significantly associated with sex (P < 0.001) and substance dependence (P = 0.004), with men more likely to have intermediate or high risk and those with substance dependence more likely to have intermediate risk. No differences were observed in the distribution of risk categories by need level (P = 0.873), psychotic disorder (P = 0.201), ethnicity (P = 0.117) or access to family physician (P = 0.242).

Table 3 show the results of both univariate and multivariate binary logistic regression examining the role of need level, diagnosis of psychotic disorder, sex, ethnicity, access to family physician and diagnosis of substance dependence in predicting the level of 30-year risk in our sample (low vs. higher risk). In univariate tests, male sex and diagnosis of substance dependence were both associated with increased CVD risk (OR = 3.99, 95% CI 2.47 to 6.56, P < 0.001, and OR = 1.94, 95% CI 1.23 to 3.06, P = 0.004, respectively) while being from an ethno-racial group was associated with reduced CVD risk (OR = 0.62, 95% CI 0.39 to 0.97, P = 0.040). In multivariate analyses, only sex and substance dependence diagnosis were significant predictors of low vs. increased risk: males were almost five times more likely to have increased CVD risk, compared to females (OR = 4.71, 95% CI 2.76 to 8.05, P < 0.001), while those with a diagnosis of substance dependence diagnosis were nearly twice more likely to have increased CVD risk compared to those without this diagnosis (OR = 1.78, 95% CI 1.05 to 3.00, P = 0.032).

**Table 3:** **Logistic Regression examining the relationship between level of 30-year CVD risk (low vs. intermediate/high) and participant level of need for mental health services, diagnosis of psychotic disorder, sex, ethnicity, access to a family physician and diagnosis of substance dependence** ^**1**^

	**Unadjusted odds ratio**		**Adjusted odds ratio**	
	**OR (95% CI)**	**P-value**	**OR (95% CI)**	**P-value**
**Variables**				
Need Level	0.89 (0.56, 1.41)	0.61	0.95 (0.54, 1.65)	0.84
Diagnosis of Psychotic Disorder	0.72 (0.47, 1.11)	0.14	0.73 (0.43, 1.25)	0.25
Sex	3.99 (2.47, 6.56)	<0.01	4.71 (2.76, 8.05)	<0.01
Ethnicity	0.62 (0.39, 0.97)	0.04	0.76 (0.45, 1.30)	0.36
Access to Family Physician	1.17 (0.75, 1.82)	0.50	1.37 (0.83, 2.26)	0.21
Diagnosis of Substance Dependence	1.94 (1.23, 3.06)	<0.01	1.78 (1.05, 3.00)	0.03

^1^Variables were dichotomized as follows: level of need for mental health services (0 = moderate need, 1 = high need); diagnosis of psychotic disorder (0 = absent, 1 = present); sex (0 = female, 1 = male); ethnicity (0 = non ethno-racial, 1 = ethno-racial); access to family physician (0 = absent; 1 = present); and diagnosis of substance dependence (0 = absent; 1 = present). The reference category was set to 0 for all variables.

## Discussion

This study found that 30-year CVD risk in a sample of homeless individuals with mental illness was more than double the level that would be achieved if their cardiovascular risk factors were optimized. White ethnicity, male sex and diagnosis of substance dependence were associated with increased 30-year CVD risk scores, but no associations were observed with level of need for mental health services, diagnosis of psychotic disorder or access to family physician. However, in adjusted analyses, only male sex and diagnosis of substance dependence were significant predictors of increased CVD risk. High need level for mental health services was associated with increased alcohol use and lower perceived stress, and a larger mean waist-to-hip ratio. Diagnosis of psychosis was associated with reduced rate of self-reported hypertension and alcohol use and lower perceived stress. White ethnicity was associated with increased rates of smoking, alcohol and cocaine use, while male sex was associated with an increased rate of smoking, marijuana use, lower BMI values, larger waist-to-hip ratios and reduced rate of hypertension, compared to female sex. Access to primary care was associated with increased hypertension, but no other predictors. Not surprisingly, diagnosis of substance dependence was associated with increased prevalence of alcohol, cocaine and marijuana use and smoking, in addition to increased perceived stress.

Previous studies of CVD risk in homeless populations have similarly observed a high prevalence of CVD risk factors and an increased risk for CVD among homeless individuals [14,28,42,43]. However, many of these studies have been limited in scope, often focusing on particular subsets of the homeless population and/or examining only one risk factor [16,44-47], or conducted outside of North America [42,43,48,49].

Two previous North American studies [14,28] have reported on CVD risk factors in addition to 10-year CHD risk in homeless males using Framingham risk calculators [14,28]. The first examined shelter-living homeless adults living in Toronto [14], who had increased prevalence of smoking as well as poor diagnosis and treatment of hypertension, hypercholesteremia and diabetes compared to the general population, but 10-year CHD risk in males from this sample did not differ from that of males from the Framingham population [14]. In the second study [28], homeless males residing in a shelter in Philadelphia did not have increased 10-year CHD Framingham risks scores compared to a group of low socioeconomic community dwelling participants, although the prevalence of both smoking and hypertension were significant higher in the homeless population [28].

Among the modifiable CVD risk factors, high rates of smoking are consistently reported in studies of both homeless and mentally ill populations [13,14,42,43,50-52]. An estimated 73 to 80% of homeless adults smoke [53,54]; tobacco companies have targeted people who are homeless in past intensive marketing strategies, including distributing free cigarettes at homeless shelters [55]. Focus group findings indicate that smoking is universal and socially acceptable in homeless settings, and many individuals smoke due to high levels of boredom and stress [56]. Similarly, individuals with a diagnosable mental illness are more than twice as likely to smoke cigarettes as the general population [57,58], with estimated prevalence rates of smoking ranging between 45 to 88% among individuals with schizophrenia, 58 to 90% among individuals with bipolar disorder, 37 to 73% among people with major depressive disorder, compared to a rate of about 20% in the general population [59]. Our study is consistent with these findings, with more than two-thirds of our sample reporting being daily or occasional smokers. Smoking rates showed significant differences by sex and ethnicity in our study; being male and non-ethno-racial were both associated with higher rates of smoking. Given that smoking represents a key modifiable CVD risk factor, primary care providers should be aware of these associations.

Interestingly, we observed limited associations with perceived stress and CVD risk in our population. In particular, we noted no association between perceived stress scores and the estimated 30-year CVD risk scores, however, perceived stress scores were higher among those with moderate need level for mental health services and a diagnosis of substance dependence, while levels were lower among those with a diagnosis of psychosis. Our participants did demonstrate higher perceived stress values compared to those from a 2009 national survey of the US general population using the same instrument (10-item PSS) (22 vs. 16, respectively); however, this finding is not unexpected given the increased stressors faced by this population [60]. A meta-analysis of prospective observational cohort studies found that high perceived stress was associated with an aggregate risk ratio of 1.27 (95% CI 1.12 to 1.45) for incident CHD, although this estimate was based only on six articles [61]. Interestingly, a recent study examining perceived stress and coronary death or non-fatal MI in the Whitehall II prospective cohort, noted that after 18 years “perceived impact of stress on health” predicted CVD risk independently of overall “perceived stress”, suggesting that the individuals’ perception of the health effects of stress may mediate the association between stress and CVD risk [62], a finding that may warrant addition of this variable in future studies. It is also important to emphasize that while previous studies report associations between levels of perceived stress and CVD risk [61,63], and several pathways of action have been proposed [63], the mechanism by which stress increases CVD risk remains unclear.

It is noteworthy that in our study, ethno-racial participants had a significantly lower 30-year CVD risk score compared to participants of non-ethno-racial ethnicity. Differences in CVD risk and risk factors by ethnicity have been reported by previous studies, with most indicating higher CVD risk among individuals of ethno-racial ethnicity [64,65], including the Canadian-based Study of Health Assessment and Risk in Ethnic groups (SHARE), which reported highest CVD rates among South Asian participants compared to participants of European and Chinese ethnicity [66]. Interestingly, a cross-sectional study of ethnic minorities in the United Kingdom using primary care practices observed the lowest CHD risk in people of African origin (7%, 95% CI 6.5 to 7.5%), compared to people of European (8.8%, 95% CI 8.2 to 9.5%) and South Asian ethnicity (9.2%, 95% CI 8.6 to 9.9%), applying the Framingham 10-year CHD estimates [67]. However, in adjusted analyses, ethnicity was no longer a significant predictor of increased CVD risk in the current study, indicating that other factors, including sex and diagnosis of substance dependence, were likely moderating this relationship in our sample. In addition, both the calculated and reference “normal” CVD risk values showed significant differences based on ethnicity in our sample, further indicating that factors other than ethnicity are responsible for this association. In the future, data on the medical care, treatment, and CVD risk profile of participants in the intervention and control groups of the study may shed light on the mechanisms underlying these differences in this sample.

Increased cardiovascular risk has been associated with frequent and heavy use of alcohol and other substances, both of which occur at high frequencies in homeless populations [68-71]. Among shelter-using adults in Toronto, 40% reported current drug problems, and marijuana, cocaine and opiates were the three most frequently used substances [68]. Cocaine in particular has been linked to a range of cardiovascular complications, including myocardial infarction, arrhythmias, sudden death and cardiomyopathy [70]. Although moderate (≤2 standard drinks/day) amounts of alcohol are associated with cardiovascular benefits, higher amounts of alcohol intake have been associated with increased CVD risk [71,72]. In our sample, diagnosis of substance dependence was a significant predictor of increased CVD risk in both unadjusted and adjusted analyses. It is of note that drug and alcohol use are not incorporated into standard CVD risk calculators, including the one used in this study.

Access to health care was suboptimal in our sample, with only 65% of all study participants reporting having a family physician. In comparison, the 2011 Canadian Community Health Survey indicated that 85% of Canadians have a regular family physician [73]. In addition, at least a third of our participants indicated not having access to care when they needed it in the past 6 months. Previous studies have also shown that homeless individuals experience barriers and difficulties in accessing health care and receiving continuity of care, even within Canada’s universal health insurance system [6,8,69,74]. However, in our study, lack of a family physician was not associated with 30-year CVD risk, and was associated only with increased risk of hypertension, likely due to possibility for diagnosis among those who receive primary care.

### Limitations

Certain limitations of this study should be noted. Study participants were recruited for a randomized controlled trial of Housing First and were not necessarily a representative sample of homeless people with mental illness. In particular, all study participants had to meet DSM-IV criteria for the presence of a mental illness at study entry; therefore our findings may not apply to facets of the homeless population who do not experience mental illness. Individuals with a prior history of mental illness, but not presenting symptoms at study entry, may have been excluded from the study. Existing clients of ACT or ICM services were also excluded, and therefore some severely ill participants already linked to services may have been excluded from our sample. Because our study only recruited legal residents of Canada, it is possible we may have missed some of the most vulnerable among homeless adults with mental illness in Toronto who do not possess legal status. Blood pressure was only measured at a study visit and diagnosis of hypertension was not confirmed by a physician. Our final sample size was reduced by the exclusion of a number of participants whose 30-year CVD risk could not be calculated. Self-reported data on CVD risk factors, as used in this study, may differ from data on risk factors obtained from medical records. Data on the lipid profile of participants were not available; however, we addressed this issue by using a CVD risk calculator that does not require information on lipid levels. We could not compare the 30-year CVD risk values with those generated by the 10-year CHD risk calculator because the latter requires lipid concentrations. While we examined differences in CVD risk between white and ethno-racial participants, we were unable to examine differences among specific ethno-racial groups because our sample had too few individuals in specific groups to conduct this analysis.

### Strengths, implications and future studies

This study adds to the literature by focusing specifically on people who are homeless and have mental illness. Clinicians who provide care for individuals who are homeless and have mental illness should be aware of the highly elevated 30-year CVD risk levels in this population, particularly among males and those who have a diagnosis of substance dependence, and make efforts to ensure appropriate risk factor modification. Future evaluation of the At Home/Chez Soi project will provide valuable information on whether a Housing First intervention can contribute to the reduction of CVD risk factors in this population.

The 30-year CVD risk calculator offers a unique opportunity to examine long-term CVD risks in individuals and in populations. Although the 10-year CHD [38,67,75] calculator is used with far greater frequency, it may not offer a long enough time-frame in which modifiable CVD risk factors can be addressed successfully to reduce CVD risks, in either individuals or populations. Greater use of the 30-year CVD risk calculator will provide us with better understanding on the efficacy and appropriateness of this tool among different populations. This study aims to add to this endeavor.

The At Home/Chez Soi project is examining how Housing First can offer a potentially viable option for addressing the unique needs of homeless individuals who experience mental illness. As longitudinal data becomes available, future studies can explore if Housing First can mitigate the increased CVD risk factors observed in this population.

## Conclusions

Homelessness is a complex social problem with many costly associated health risks, including cardiovascular disease. Homeless people with mental illness, recruited as participants at the Toronto site of the At Home/Chez Soi study, have highly elevated 30-year CVD risk, particularly among males and those diagnosed with substance dependence. This study adds to the literature by reporting on CVD risk in a particularly vulnerable population of homeless adults experiencing mental illness, and by using a 30-year CVD risk calculator which permits for longer period of time during which modifiable CVD risk factors could be improved.

## Additional file

Additional file 1:
**Supplemental Tables.**

## Abbreviations

BMI: Body mass index (kg/m^2^)
CVD: Cardiovascular disease
OR: Odds ratio
