# Supplementary material for: Cardiovascular risk factors and 30-year cardiovascular risk in homeless adults with mental illness
Source: BMC Public Health. 2015 Feb 23;15:165. doi: 10.1186/s12889-015-1472-4 (PMC4339633; doi:10.1186/s12889-015-1472-4)
Supplement: Additional file 1: — Supplemental Tables. [file 12889_2015_1472_MOESM1_ESM.docx]

**Additional Files**

**Supplemental Table 1:** Definitions for Housing Status [[33](#_ENREF_33)].

| **Housing Status** | **Definition** |
| --- | --- |
| Absolutely homeless | no fixed place to stay for at least the past 7 nights with little likelihood of finding a place in the upcoming month |
| Precariously housed | housed in single room occupancy (SRO), rooming house, or hotel/motel as a primary residence AND in the past year have a history of 2 or more episodes of being Absolutely Homeless OR one episode of being absolutely homeless of at least 4 weeks duration in the past year |
| Relatively homeless | individuals who inhabit spaces that do not meet the basic health and safety standards, such as living in overcrowded or hazardous conditions |

**Supplemental Table 2:** Classification of High and Moderate Need^a^

| **Need Level** | **Criteria** |
| --- | --- |
| High | score of ≤62 on the Multnomah Community Ability Scale (MCAS)  A Mini International Neuropsychiatric Interview (MINI) diagnosis of current psychotic disorder or bipolar disorder or an observation of psychotic disorder on the screener  AND one of:  ≥2 hospitalizations for mental illness in any 1 year of the past 5 years  Comorbid substance use (based on MINI)  Recent arrests of incarcerations |
| Moderate | All other participants who have met eligibility criteria but do not meet the criteria above for High Need Level |

^a^ For more details, please see [[33](#_ENREF_33)]

**Supplemental Table 3:** Variables necessary for “office” calculator of 30-year CVD risk [[32](#_ENREF_32)].

| **Variable** | **Description of Variables** | **Ranges/units** |
| --- | --- | --- |
| SEX | Sex; m or f | m or f |
| AGE | Age in years | >=20 and <60 |
| SBP | Systolic blood pressure | 78-240 mmHg |
| SMOKE | Smoking | y or n |
| TRTBP | Treatment for high blood pressure | y or n |
| BMI | Body mass index | kg/m2 |
| DIAB | Diabetes | y or n |

**Supplemental Table 4:** Calculated CVD Risk and estimated normal CVD risk by level of need for mental health services, diagnosis of psychotic disorder, sex, ethnicity, access to a family physician and diagnosis of substance dependence**.**^a^

|  | **Need Level** | | | **Diagnosis of Psychotic Disorder^b^** | | | **Sex** | | | **Ethnicity** | | | **Access to Family Physician** | | | **Diagnosis of Substance Dependence** | | | |
| --- | --- | --- | --- | --- | --- | --- | --- | --- | --- | --- | --- | --- | --- | --- | --- | --- | --- | --- | --- |
|  | **Moderate** | **High** | ***P*^c^** | **Yes** | **No** | ***P*^c^** | **Males** | **Females** | ***P*^c^** | **Ethno- Racial** | **Non Ethno- Racial** | ***P*^c^** | **Yes** | **No** | ***P*^c^** | **Yes** | **No** | ***P*^c^** |  |
|  | **(N=243)** | **(N=109)** |  | **(N=134)** | **(N=218)** |  | **(N=250)** | **(N=102)** |  | **(N=217)** | **(N=119)** |  | **(n=227)** | **(n=122)** |  | **(N=128)** | **(N=224)** |  |  |
| **30- year Hard CVD Risk Scores (%)** | | | | | | | | | | | | | | | | | | |  |
| Calculated | 21.6 ± 18.2 | 21.2 ± 18.8 | 0.876 | 18.9 ± 16.6 | 23.0 ± 19.3 | 0.12 | 25.2 ± 18.8 | 12.2 ± 13.4 | <0.01 | 20.0 ± 18.7 | 23.7 ± 17.1 | 0.01 | 22.6 ± 19.0 | 19.6 ± 17.3 | 0.14 | 22.5 ± 16.4. | 20.9 ± 19.5 | 0.42 |  |
| “Normal“ | 10.3 ± 7.37 | 9.58 ± 6.87 | 0.56 | 9.15 ± 6.81 | 10.6 ± 7.41 | 0.11 | 12.2 ± 13.4 | 5.32 ± 4.19 | <0.01 | 9.26 ± 6.69 | 11.8 ± 7.82 | <0.01 | 10.4 ± 7.48 | 9.52 ± 6.73 | 0.26 | 10.0 ± 6.58 | 10.1 ± 7.56 | 0.86 |  |

^a^ CVD risk was calculated using the BMI-based 30 year CVD risk algorithm proposed by Pencina et al [[32](#_ENREF_32)]. The algorithm is based on the following variables: age, sex, systolic blood pressure, BMI, current smoker (Y/N), presence of diabetes (Y/N), treatment for hypertension (Y/N).

^b^ Presence of Psychotic Disorder was assessed by the MINI International Neuropsychiatric Interview at study entry.

^c^ P-values represent group differences based on Mann-Whitney U tests (comparisons were made within groups on a particular row.
